# Mouth Rinsing with Maltodextrin Solutions Fails to Improve Time Trial Endurance Cycling Performance in Recreational Athletes

**DOI:** 10.3390/nu8050269

**Published:** 2016-05-09

**Authors:** Tuğba Nilay Kulaksız, Şükran Nazan Koşar, Suleyman Bulut, Yasemin Güzel, Marcus Elisabeth Theodorus Willems, Tahir Hazir, Hüseyin Hüsrev Turnagöl

**Affiliations:** 1 Division of Nutrition and Metabolism in Exercise, Faculty of Sport Sciences, Hacettepe University, Beytepe, Ankara 06800, Turkey; nilaygungor17@gmail.com (T.N.K.); nazankosar@gmail.com (Ş.N.K); slmbulut@gmail.com (S.B.); ysmnonur@gmail.com (Y.G.); 2 Department of Sport Sciences, Bağlıca Campus, Başkent University, Ankara 06530, Turkey; 3 Department of Sport and Exercise Sciences, University of Chichester, College Lane, Chichester PO17 6PE, UK; m.willems@chi.ac.uk; 4 Division of Sport and Coaching, Faculty of Sport Sciences, Hacettepe University, Beytepe, Ankara 06800, Turkey; thazir@hacettepe.edu.tr

**Keywords:** maltodextrin solution, mouth rinse, endurance performance, cycling

## Abstract

The carbohydrate (CHO) concentration of a mouth rinsing solution might influence the CHO sensing receptors in the mouth, with consequent activation of brain regions involved in reward, motivation and regulation of motor activity. The purpose of the present study was to examine the effects of maltodextrin mouth rinsing with different concentrations (3%, 6% and 12%) after an overnight fast on a 20 km cycling time trial performance. Nine recreationally active, healthy males (age: 24 ± 2 years; $\dot{V}O_{2max}$: 47 ± 5 mL·kg^−1^·min^−1^) participated in this study. A double-blind, placebo-controlled randomized study was conducted. Participants mouth-rinsed every 2.5 km for 5 s. Maltodextrin mouth rinse with concentrations of 3%, 6% or 12% did not change time to complete the time trial and power output compared to placebo (*p* > 0.05). Time trial completion times were 40.2 ± 4.0, 40.1 ± 3.9, 40.1 ± 4.4, and 39.3 ± 4.2 min and power output 205 ± 22, 206 ± 25, 210 ± 24, and 205 ± 23 W for placebo, 3%, 6%, and 12% maltodextrin conditions, respectively. Heart rate, lactate, glucose, and rating of perceived exertion did not differ between trials (*p* > 0.05). In conclusion, mouth rinsing with different maltodextrin concentrations after an overnight fast did not affect the physiological responses and performance during a 20 km cycling time trial in recreationally active males.

## 1. Introduction

Carbohydrate (CHO) consumption during prolonged (>2 h) exercise improves exercise performance by maintaining plasma glucose levels, providing fuel for the working muscles and sparing muscle glycogen stores [1,2]. In this regard, an increased time to fatigue by 33% was observed during cycling at 71% $\dot{V}O_{2max}$ in highly trained endurance cyclists ($\dot{V}O_{2max}$ = 66.9 ± 1.3 mL·kg^−1^·min^−1^) who were accustomed to exercising for prolonged periods (2–4 h), when fed a CHO solution [3]. Similarly, CHO intake by endurance-trained men during exercise at an intensity of 69% $\dot{V}O_{2max}$ increased exercise time to exhaustion by about 30% [4]. However, it has been shown that CHO consumption during high intensity endurance exercise (<1 h) also resulted in improved performance [5], which cannot be explained by the metabolic effects. Indeed, in the first hour of high intensity endurance exercise (~80% $\dot{V}O_{2max}$), only 5 to 15 g of the consumed CHO was estimated to be oxidized [5] and too small to improve exercise performance compared with the total amount of ingested CHO [5].

The absence of a metabolic explanation for the ergogenic effects of CHO ingestion during high intensity endurance exercise has lead researchers to consider a non-metabolic mechanism. In an attempt to bypass taste and CHO receptors in the mouth and inter-individual variations in absorption rate, Carter *et al.* [6] examined the effects of glucose infusion on a time trial of 1 h exercise. No improvement was observed for the 1 h time trial performance in spite of increased availability of plasma glucose for oxidation and increased glucose uptake into the tissues [6]. These findings may indicate that ergogenic effects of exogenous CHO during high intensity endurance cycling (~75% $\dot{\text{V}}O_{2max}$) and short duration endurance exercise (~1 h) are regulated by central non-metabolic mechanisms. Potential central non-metabolic mechanisms of CHO mouth rinse were first examined by Carter *et al.* [7]. Endurance cyclists completed a set amount of work (*i.e.*, a 1 h cycling time trial) with CHO mouth rinsing (*i.e.*, 6.4% maltodextrin) every 12.5% of the trial and improved performance time by 2.8% [7]. The study by Carter *et al.* [7] provided the first observation on the beneficial effects of CHO mouth rinse and improvement of short duration endurance performance.

Carbohydrate mouth rinse studies raised the possibility that ergogenic effects were due to activation of certain brain regions by CHO presence in the mouth. To test this, Chambers *et al.* [8] determined the brain regions activated by CHO mouth rinse using functional magnetic resonance imaging (fMRI). Endurance trained cyclists rinsed their mouth with either 6.4% glucose solution, 6.4% maltodextrin or placebo solution containing saccharin. Both CHO solutions reduced the time to complete the cycle time trial and activated similar pattern of brain regions compared with placebo. Brain regions activated by the presence of CHO in the mouth were the areas of the insula/frontal operculum, orbitofrontal cortex and striatum [8], which are believed to be involved in reward, motivation and regulation of motor activity. Furthermore, more brain regions are activated in response to CHO compared to artificial sweeteners [8,9] suggesting that it is not the taste of the solution but the presence of CHO in the mouth that seems to be associated with exercise performance enhancements by CHO rinse. Therefore, the CHO content of the rinsing solution may exert a dose dependent effect on exercise performance.

Although there is a growing number of research papers on the effects of CHO rinse on high intensity endurance exercise performance [7,8,10,11,12,13,14,15,16,17,18,19,20], cycling sprint performance [21,22] and, neuromuscular function [23,24] most of the CHO rinse studies investigated the effects of with approximately 6% CHO solutions. Recently, Ispoglou *et al*. [20] compared the effectiveness of mouth rinsing with varying CHO content (4%, 6%, and 8% solutions) on 1-h simulated cycling time trial performance in the postprandial state which failed to improve performance.

Although inconclusive, several studies [10,11,18] showed that performance benefits of CHO mouth rinsing increases with participants in the fasted state compared to post-absorptive or postprandial states, which has been further proved by a functional magnetic resonance imaging (fMRI) study [9] revealing that after the 12 h fasting period more brain regions were activated by sucrose compared with the fed state. Therefore, the primary aim of the present study was to examine the effects of mouth rinsing with different concentrations of maltodextrin (MD) solutions (*i.e.*, 3%, 6% and 12%) on 20 km cycling time trial endurance performance after an overnight fast. We hypothesized that a dose-dependent performance effect would be observed with increased MD content compared to a non-CHO placebo. Most of the CHO rinse studies on endurance cycling performance were conducted with competitive [11], trained [7,8,12,17,18,20] or recreational cyclists [16]. Studies on non-athletes [10] or physically active non-cyclists are sparse [23]. Since the effect of mouth rinse is achieved through CHO sensing receptors and thus stimulation of reward centers in the brain and the practices performed by the trained athletes may be adopted by recreational athletes, it is of interest to determine if maltodextrin mouth rinse was effective in non-cyclist recreational athletes as well.

## 2. Materials and Methods

### 2.1. Participants

Nine recreationally active healthy males (age: 24 ± 2 years, body mass: 80 ± 11 kg, body mass index: 25.2 ± 2.7 kg·m^−2^, body fat: 21.8% ± 3.3%, maximum cycling power (W_max_): 280 ± 39 W, predicted maximum oxygen consumption ($\dot{V}O_{2max}$): 47 ± 5 mL∙kg^−1^∙min^−1^) volunteered in this study. Participants were informed of the nature and possible risks of the study and provided written consent. Hacettepe University Non-interventional Clinical Research Ethics Board approved the experimental protocol (decree no: LUT 12/135-11).

### 2.2. Experimental Design

The study had a double-blind, placebo-controlled, randomized experimental design. Participants visited the laboratory five times. In brief, during the first visit, participants performed an incremental cycling protocol. In each of the remaining four visits, participants were instructed to cycle 20 km with 2.5 kg resistance as fast as possible with provision of a 3%, 6%, or 12% MD solution or placebo (PLA) mouth rinse at 2.5 km intervals. Cycling trials were separated by at least 48 h and exercise sessions took place at the same time of the day (9–11 a.m.) after a 10 h overnight fast. Participants were asked to record their dietary intake before the visits and to abstain from caffeine, alcohol, and strenuous exercise for the 24 h preceding an experimental trial. All tests were carried out on a mechanically braked cycle ergometer (Monark Ergomedic 834 E, Varberg, Sweden).

### 2.3. Preliminary Testing

During the first visit, anthropometric and body composition measurements were taken followed by an incremental cycling protocol to exhaustion to determine maximum cycling power at exhaustion (W_max_) and predict $\dot{V}O_{2max}$. The protocol was similar to Storer *et al.* [25]. Briefly, after a warm up (4 min at 0 W), the incremental cycling protocol was initiated with a starting power of 60 W for one minute followed by increments of 15 W per min until exhaustion with participants instructed to keep pedal revolution at 60 rpm. Achievement of at least two of the following criteria was used to verify that a true maximum test was performed: Percentage of age predicted maximum heart rate > 90%, pedal revolution <60 rpm, rating of perceived exertion (RPE) ≥18 (Borg’s Scale with rating of 6–20) [26]. Maximum cycling power at exhaustion was determined using the following equation:

(1) $$W_{\text{max}}=W_{\text{out}}\;+\;\left(t/60\right)\;\times \;15$$

With *W*_max_ maximum cycling power at exhaustion, *W*_out_ the last workload of the completed stage, *t* the time of the final unfinished stage, 60 the time in seconds between two stages and 15 the workload increase between stages.

Maximum oxygen uptake of the participants was calculated according to the equation (*R* = 0.939, SEE = 2.57 mL∙kg^−1^∙min^−1^) by Storer *et al.* [25]:

(2) $$\dot{V}O_{2\text{max}}=10.51\;\left(W_{\text{max}}\right)\;+\;6.35\;\left(\text{body mass}\right)\;-\;10.49\;\left(\text{age}\right)+519.3\text{ mL}\cdot {\text{min}}^{-1}$$

With $\dot{V}O_{2max}$ maximum oxygen uptake and *W*_max_ maximum cycling power at exhaustion. Body mass and age are expressed in kg and years, respectively.

### 2.4. Experimental Trials

On arrival at the laboratory, resting heart rate was recorded for 5 min and resting blood lactate (YSI 1500; Yellow Springs Instruments; Yellow Springs, OH, USA) and glucose (One Touch Select, LifeScan, Inc., Chesterbrook, PA, USA) levels were measured with blood samples taken from the fingertip. The lactate analyzer was calibrated before each test using a standard solution with a lactate concentration of 5.0 mmol·L^−1^ (allowing a measurement error within the ±2% range). Coefficient of variation for One Touch Select was reported as ≤5% or a standard deviation of ≤5 mg·dL^−1^ [27].

**Figure 1:**
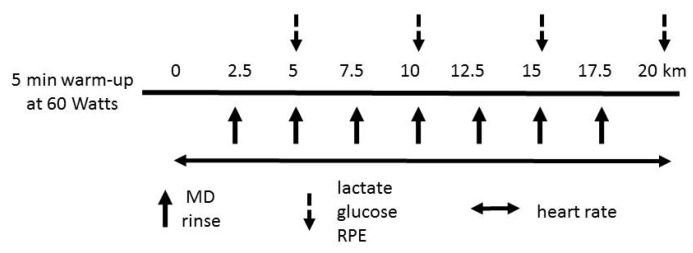
Outline of tests and testing procedures. MD: maltodextrin, RPE: rating of perceived exertion.

For the experimental trials, participants performed a warm up for 5 min at 60 W. After the warm up, participants cycled a 20 km distance as fast as possible with 2.5 kg (150 W) constant resistance. We decided on a 20 km time trial as preliminary work showed our participants to be able to complete approximately 20 km of distance in less than 1 h at 65% to 80% *W*_max_. Since a mechanically braked cycle ergometer (Monark Ergomedic 894E, Varberg, Sweden) was used in the present study, it was not possible to observe the completed amount of work instantly. Therefore, distance (20 km) and resistance (2.5 kg) were maintained constant to ensure that each participant performed an equal amount of work (*i.e.*, 495 kJ). During the 20 km time trials, participants rinsed their mouth with different concentrations of MD solutions or placebo solution at 2.5, 5, 7.5, 10, 12.5, 15 and 17.5 km of the trial. The distance covered was displayed on the ergometer. Blood lactate, blood glucose and RPE were measured every 5 km of the trial. Heart rate was recorded every 5 s using short-range telemetry (Polar 810i, Polar Instruments, Kempele, Finland) throughout each trial. The time to complete each 5 km was recorded to calculate mean power output of each 5 km period. In the present study, we did not conduct a familiarization trial, but all participants were familiar to cycling. A schematic overview of the experimental protocol is shown in Figure 1.

### 2.5. Mouth Rinse Protocol

During the experimental trials, each participant was given a 50 mL bolus of either 3%, 6% or 12% MD (Fantomalt, Nutricia, UK) solution or placebo solution (0% MD) every 2.5 km. The participants were instructed to rinse the fluid around their mouths for 5 s, and then spit the fluid into a graded container which was measured to ensure that none of the rinsing solution had been ingested. The amount of expectorated solution was the same or even higher in some cases to the amount of rinsed solution. A 5-s mouth rinse protocol was adopted in the present study as longer duration would interfere with the respiration and may result in power output decrease during the trial [18,28]. Moreover, during the pilot testing, as participants were swallowing some part of the solution, it was decided to use a more conventional approach (*i.e.*, 5 s) more appropriate for field settings. In addition, a 5-s mouth rinse allowed comparison to other relevant studies with respect to mouth rinse duration.

In the present study, MD was selected because it is colorless and nonsweet when dissolved in water. Solutions were therefore indistinguisable with each other and the placebo. MD solutions with 3%, 6%, and 12% were prepared by dissolving 15 g, 30 g and 60 g MD into 500 mL of water, respectively. To make MD and placebo solutions indistinguishable in taste, 0.5 g artificial sweetener (Aspartame and Acesulfame-K, Milchwerke “Mittelelbe” GmbH, Stendal, Germany) was added to each solution. One participant only was able to guess the correct condition of testing.

Most of the CHO mouth rinse studies in the literature, reviewed by Burke and Maughan [29], used a 25 mL bolus. It has been suggested that increased contact time of CHO with carbohydrate taste receptors might increase the activation of reward regions in the brain [16]. However, increasing the duration of rinsing may interfere with the respiration and thus reducing the power output during exercise [18,28]. Therefore, we aimed to increase contact of MD with carbohydrate sensing receptors by increasing the amount of rinsed solution. Therefore, we selected to use a 50 mL bolus.

### 2.6. Dietary Procedures

Participants recorded their diet in the 24-h period before each experimental trial to assess dietary intakes prior to each trial. Participants were instructed to replicate the diet prior to every trial and consume enough water throughout the study. Dietary records were analyzed with the Turkish Nutrition Data System (BEBIS 6.1, Hohenheim University, Stuttgart, Germany) software to quantify total energy intake (kcal), fat, CHO, protein and water consumption.

### 2.7. Statistical Analyses

Sample size was calculated using G*Power software (version 3.1.9.2, Franz Faul, Universitat Kiel, Dusseldorf, Germany) for Repeated Measures ANOVA for detecting a medium effect size (Cohen’s *d* = 0.5) with α as 0.05 and power of study as 90%, which revealed that a sample size of 9 participants was needed. One-way repeated measures analysis of variance (RM-ANOVA) was used to compare the effects of mouth rinsing with different concentrations of MD solutions on 20 km time trial performance as well as resting physiological variables and nutrient intakes, and to assess whether there was a learning effect for performance data. All measures taken during the time trial at every 5 km were compared using 4 × 4 (Condition (placebo, 3%, 6%, and 12% MD mouth rinse) × Time (0–5, 5–10, 10–15, 15–20 km distance intervals)) RM-ANOVA to determine whether there was a main effect of time or condition and condition × time interaction effect on heart rate, lactate, glucose, RPE, mean power output and time to complete each 5 km distance interval. Mauchly test was used to determine the sphericity assumption of the repeated measures. When sphericity assumption was violated Greenhouse-Geisser and Huynh-Feldt corrections were applied if epsilon (ε) is <0.75 and ≥0.75, respectively [30]. Magnitude of the size effect was reported according to Hopkins as follows [31]: If Eta square (η^2^) was 0 < η^2^ < 0.2 a small effect, 0.2 < η^2^ ≤ 0.6 a medium effect, 0.6 < η^2^ ≤ 1.2 a large effect, 1.2 < η^2^ < 2.0 a very large effect, and 2.0 < η^2^ < 4.0 near perfect. When the ANOVA revealed significant interactions, Bonferroni *post hoc* analysis was applied to compare the differences. Statistical analysis was performed by SPSS (version 16.0 IBM, Chicago, IL, USA), statistical significance level was assumed at *p* < 0.05.

## 3. Results

**Figure 2:**
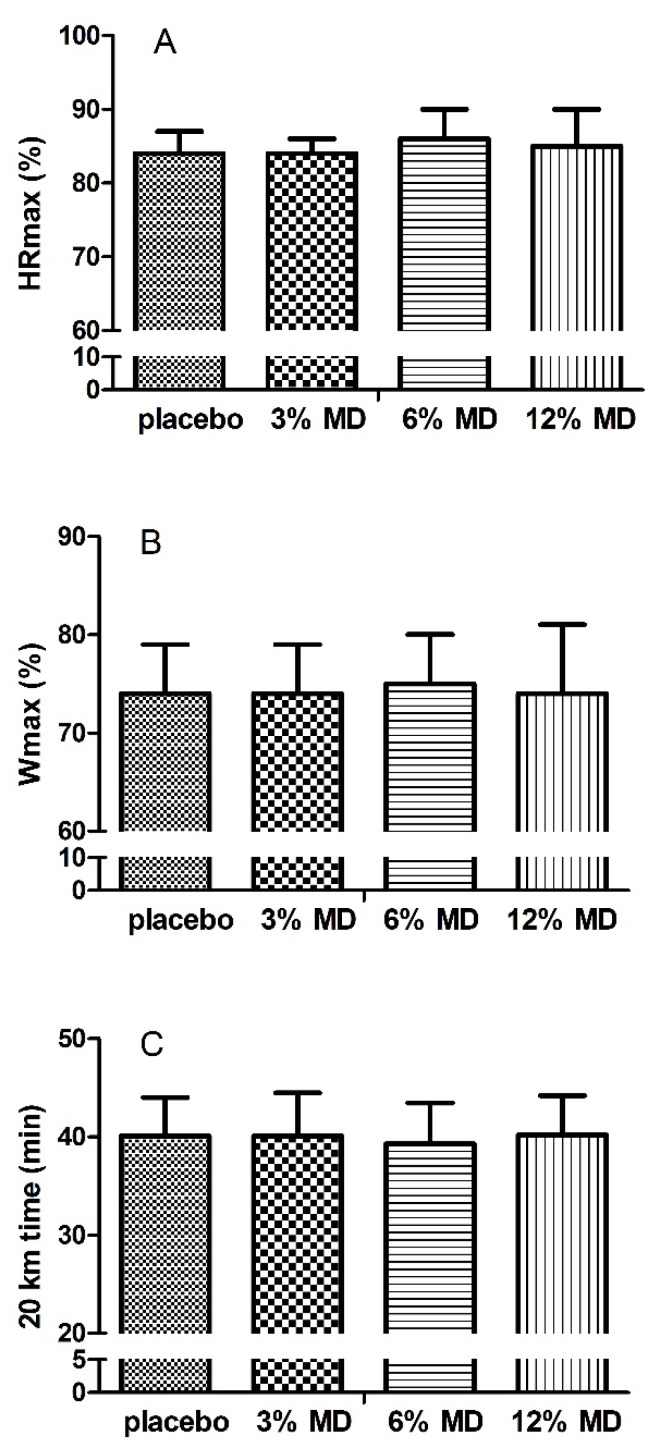
Percentages of HR_max_ (**A**); W_max_ (**B**), achieved and 20 km time trial times (**C**). MD, maltodextrin. HR_max_, maximum heart rate. W_max_, maximum cycling power.

**Figure 3:**
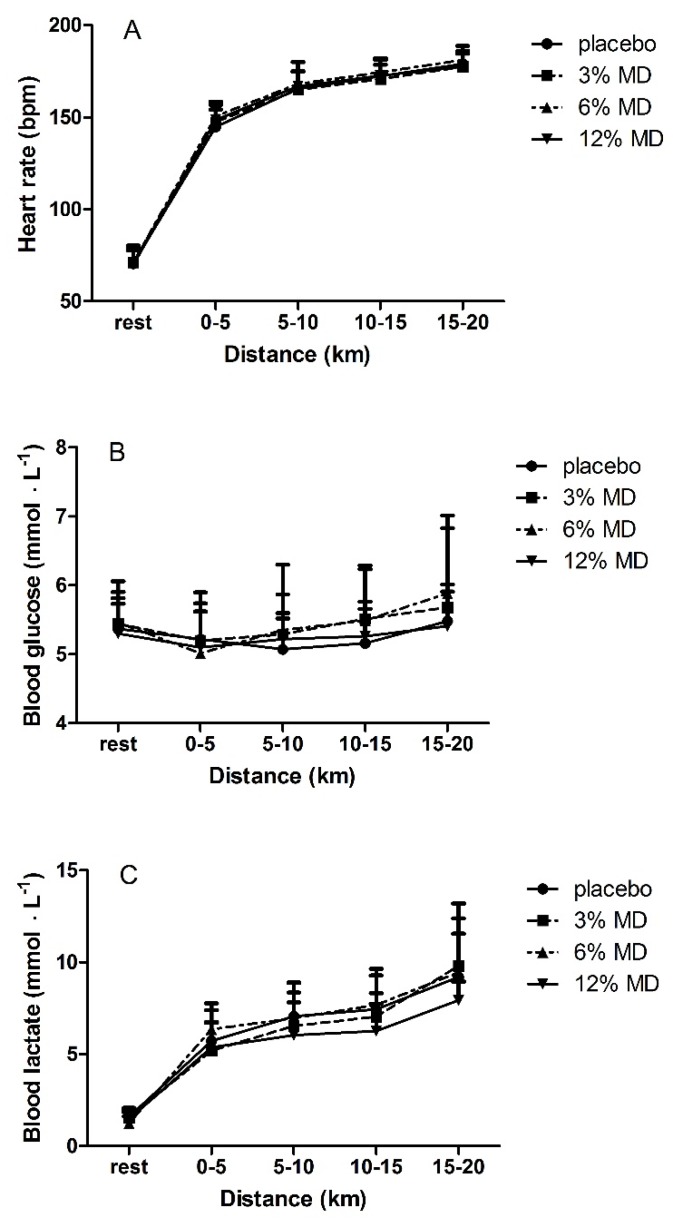
Heart rate (**A**); blood glucose (**B**); and blood lactate (**C**) as a function of cycled distance. MD, maltodextrin.

One way RM-ANOVA results for dietary analysis (Table 1), resting heart rate, blood lactate and glucose (Figure 3) revealed that resting physiological variables, total energy and macronutrient intake were similar between trials (*p* > 0.05), indicating that participants initiated the trials under the same physiological and nutritional states. Furthermore, at each trial, participants exercised at ~65% to 80% W_max_ (*F* = 1.083, *p* > 0.05, Figure 2) and on average 85% of predicted maximum heart rate (HR_max_, *F* = 0.774, *p* > 0.05, Figure 2). The trial completion times for MD solutions with 3%, 6% and 12% (*i.e.*, 40.07 ± 3.92, 40.08 ± 4.39, 39.25 ± 4.18 min, respectively) and placebo (40.18 ± 4.00 min) were similar (*F* = 1.094, *p* > 0.05, Figure 2) indicating no improvement in time to complete the trial with mouth rinsing with MD solutions. In addition, time trial performance times of consecutive trials were similar (*F(*3, 32) = 0.453, *p* = 0.717) excluding a learning effect in the absence of familiarization.

No significant interaction effect for trial x time for blood lactate, glucose and heart rate (*F(*1.99, 7.96) = 0.465, *F(*2.9, 23.3) = 1.191 and *F(*2.92, 20.51) = 0.67, respectively, *p* > 0.05) (Figure 3) or main effect of trial (*F(*3, 12) = 0.931, *F(*3, 24) = 1.072 and *F(*3, 21) = 0.768, respectively, *p* > 0.05) was detected indicating that these physiological responses to the 20 km time trial were similar among the MD rinsing trials and placebo. However, a significant main effect for time was observed for lactate, glucose and heart rate (*F(*1.08, 4.32) = 13.31, *F(*1.6, 12.8) = 10.48 and *F(*1.36, 9.54) = 111.743, respectively, *p* < 0.05) indicating that all these variables increased during each trial (Figure 3). Bonferroni *post hoc* analysis showed that heart rate increased significantly every 5 km distance (*p* < 0.05, Figure 3). Glucose levels at 10–15 km and 15–20 km distance periods were significantly higher than at 0–5 km (*p* < 0.05, Figure 3). Lactate levels at 5–10 km and 10–15 km distance periods were significantly higher compared to 0–5 km distance period (*p* < 0.05, Figure 3).

**Figure 4:**
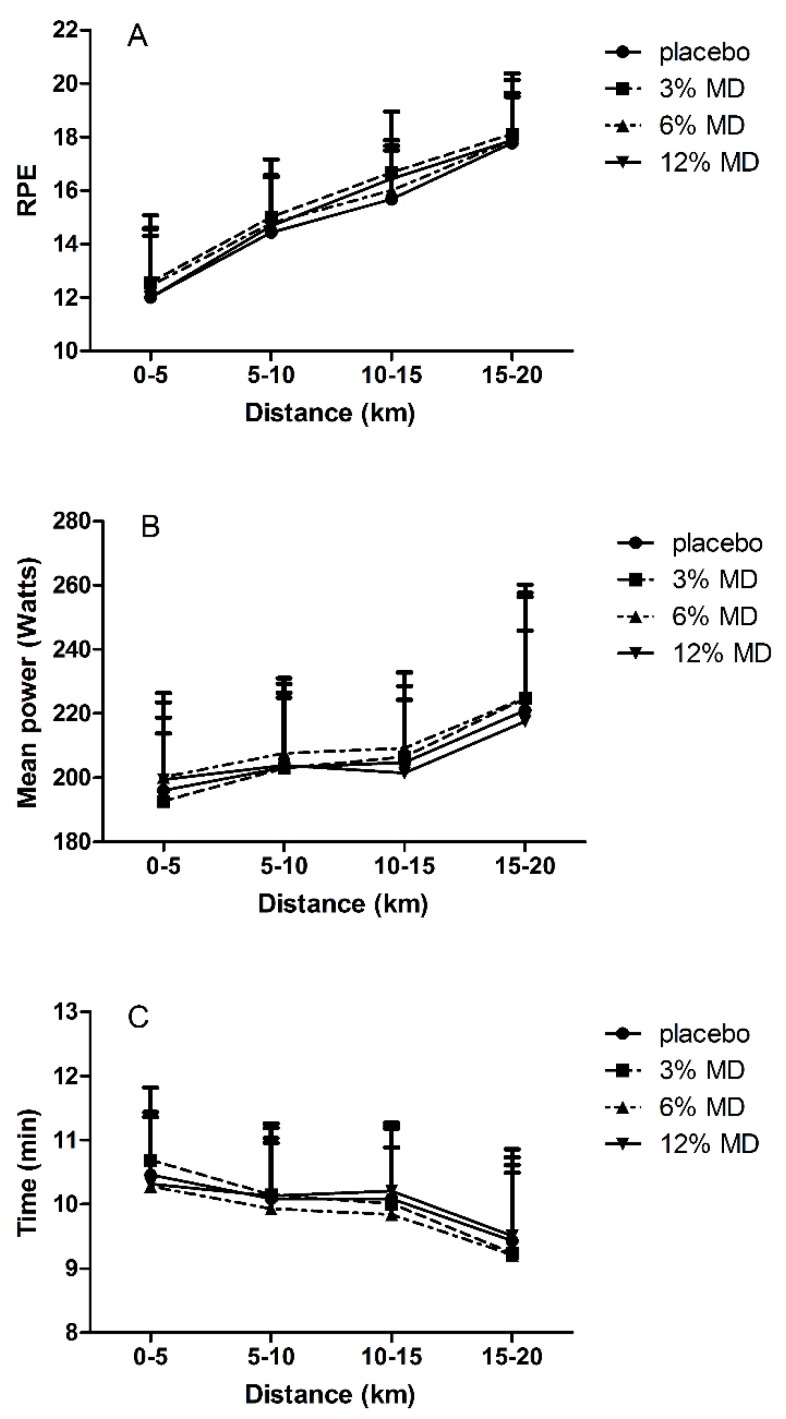
RPE (**A**), mean power (**B**); and time to complete distance intervals (**C**) as a function of cycled distance. MD, maltodextrin. RPE, rating of perceived exertion.

Two way RM-ANOVA results showed no interaction effect of trial × time (*F(*2.44, 19.54) = 1.642, *F(*2.01, 16.01) = 1.612 and *F(*2.9, 23.3) = 1.191, respectively, *p* > 0.05) (Figure 4) or main effect of trial (*F(*3, 24) = 1.094, *F(*3, 24) = 0.984 and *F(*3, 24) = 0.619, respectively, *p* > 0.05) on time to complete 5-km distances, average power and RPE. These findings indicated similar pacing strategy, average power output and RPE responses to mouth rinsing with different MD concentrations or placebo solutions on these parameters. Significant main time effects were observed for time to complete 5 km distance periods, average power output and RPE values (*F(*1.12, 8.95) = 7.545, *F(*1.06, 8.52) = 6.657 and *F(*1.22, 9.8) = 28.87, respectively, *p* < 0.05) which indicated that the time to complete each 5 km distances decreased with increases of average power and RPE (Figure 4). *Post hoc* analysis revealed that RPE increased with distance covered (*p* < 0.05, Figure 4). Mean power increased accompanying with decrease in time to complete distance intervals from 0–5 km to 5–10 km, and from 10–15 km to 15–20 km (*p* < 0.05, Figure 4).

## 4. Discussion

The purpose of this study was to determine if there is a dose dependent effect of mouth rinsing with maltodextrin solutions on a 20 km cycling time trial in the fasted state in recreational athletes. We observed that maltodextrin mouth rinse with concentrations of 3%, 6% or 12% did not change time to complete the time trial and power output compared to placebo (*p* > 0.05). In addition, heart rate, blood lactate, blood glucose, and RPE did not differ between trials (*p* > 0.05). These findings therefore indicate that mouth rinsing with maltodextrin solutions of varying concentrations does not improve endurance cycling time trial performance in recreational athletes. A study by Ispoglou *et al*. [20] compared the effect of 4%, 6%, and 8% of CHO solutions on a 1 h simulated cycling trial in the postprandial state and observed no improvement with either concentration of CHO solutions compared to a non-CHO placebo. The present study was the first comparing more diverse (hypotonic, isotonic and hypertonic) mouth rinse MD solutions (3%, 6%, and 12%) in the fasted state on exercise performance. Use of maltodextrin, which is colorless and tasteless when dissolved in water, allowed us to make the solutions indistinguishable from each other and the placebo, as well as to determine the specific ergogenic effect of maltodextrin rinse but not any other substance.

In a recent review by Burke and Maughan [29], it was concluded that the benefits of CHO mouth rinse was achievable by frequent (every 5–10 min) and significant contact between oral cavity and a carbohydrate source, independent of a sweet taste. The rinsing frequency in the present study was 5.70 ± 0.59 min which is at the most frequent side of the recommended range. In contrast to most of the studies (reviewed in [29]), we used a 50 mL bolus to increase the contact between CHO source and the CHO sensing receptors in the mouth without increasing the rinsing duration which has been suggested to interfere with the rhythm of the respiration and thus decrease power output during exercise [18,28]. However, the findings of the present study revealed that mouth rinsing with different concentrations of MD after an overnight fast failed to improve exercise performance compared to a non-CHO placebo.

Although other studies [15,17,19,20] also reported no endurance performance improvement with CHO rinse, there is abundant evidence that CHO mouth rinsing improves endurance exercise performance [7,8,10,11,12,13,14,15,16,32]. Indeed, the overall effect of CHO mouth rinse on performance was found to be significant (mean difference = 5.05 W, 95% CI 0.90 to 9.2 W, *z* = 2.39, *p* = 0.02) [32]. However, a large variation was observed between the studies (*I*^2^ = 52%) [32], which has been attributed to methodological differences; participants’ nutritional state (fasting, postprandial or post absorptive state), duration of the mouth rinse (5 s or 10 s), mouth rinsing frequency (recommended every 5 to 10 min), type of activity (running *vs*. cycling), exercise protocols and sample size [29,32]. Another factor for consideration is the type of placebo or control group (water rinse, artificially sweetened non-CHO solution or no rinsing at all) with which the ergogenic effect of CHO rinse were compared [18,33]. Therefore, our discussion is mainly focused on the methodological differences between the present study and the CHO rinse studies determining endurance cycling performance changes [7,8,11,16,17,18,33].

Most of the CHO mouth rinse studies on cycling endurance performance used maltodextrin solutions of 6% and 6.4% [7,8,16,17,18] and 10% [11]. Among these studies, only one [17] reported no performance improvement with MD mouth rinse, two were conducted in the fasting state [8,11] while others at postprandial or post absorptive states [7,16,17,18]. Similar to our study, two studies [8,11] compared the MD rinse performance with an artificially sweetened placebo while others used water rinse [7,16,17,18], and one no rinse control [17]. With respect to nutritional state and type of placebo group, our study was similar to studies by Chambers *et al.* [8] and Lane *et al.* [11]. In addition, a 10% MD mouth rinse solutions was used [8,11], which is the closest concentration to the 12% MD of the present study. However, the rinsing duration was longer, competitive well trained cyclists served as participants and the duration of trial was longer (~60 *vs*. 40 min) in those studies [8,11] compared to the present study. By giving a 50 mL bolus, it was intended to increase the contact between CHO source and the CHO sensing receptors in the mouth without increasing the rinsing duration. Therefore, the only factor that may explain the differences in performance outcomes was the fitness levels of the participants. Although most of the CHO rinse studies on endurance cycling performance were conducted with competitive [11], trained [7,8,12,17,18,20,33] or recreational cyclists [16], Fares and Kayser [10] investigated the effects of CHO mouth rinse on cycling performance at different fasting states in a nonathletic participants (mean $\dot{V}O_{2max}$: was 31 ± 7 mL∙kg^−1^∙min^−1^). Fares and Kayser [10] reported improved performance with CHO mouth rinsing in contrast to our findings. Maximum oxygen uptake values of our participants were 47 ± 5 mL∙kg^−1^∙min^−1^. Most of the studies in the literature, $\dot{V}O_{2max}$ values of the participants were higher (~21% to 42%) than in our study. Some of these studies reported improvement in cycling exercise performance [7,8,11,12], although others report no improvement [17]. This may explain the differences between the present results and those published previously.

Perception of pleasantness and thus the activation level of brain reward regions seems to increase under extreme conditions (*i.e.*, fatigue, dehydration, fasting) [10,11,18,23,24,33]. Blood lactate, heart rate, RPE and percentage of maximum power outputs during the trials seem to indicate that participants in the present study maximized effort. Therefore, effort cannot be accounted for the failure to find a performance improvement with any MD solution in the present study.

With regard to nutritional intakes prior to the day of the experiment and recovery of glycogen stores between the trials, dietary analysis data showed that nutritional status were similar prior to each trial implying that participants performed the trials at identical nutritional states. Relative values for protein and carbohydrate indicated that participants consumed the recommended daily amounts. In addition, we did not observe decrease in glucose levels which further supports recovery of the participants between the trials. Therefore, this may not be the reason for the differences in performance changes between our study and others [7,8,11,16,18] reporting improved cycling endurance performance.

Primary finding of the present study was that trial completion time did not improve with any of the MD rinse conditions compared to non-CHO placebo group which is in agreement with the findings of Che Muhammed *et al.* [33] in that no difference was found between CHO and non-CHO mouth rinsing conditions in cycling time trial performance although the study was conducted under more challenging conditions, *i.e.*, during Ramadan fasting in a hot-humid environment. However, a significant performance improvement was observed with both CHO and non-CHO placebo rinse compared to the no-rinse group [33]. Ispoglou *et al.* [20] reported similar findings to ours with participants in the postprandial state comparing 4%, 6%, and 8% CHO rinse with non-CHO placebo. In the present study, and the study by Ispoglou [20], absence of a non-rinse group is the main limitation that might have obscured any performance improvement with mouth rinse with either CHO or artificially sweetened solution compared to no-rinse control. Significance of the type of placebo group was evidenced by the study of Gam *et al.* [18] showing that mouth rinse with water decreased cycling time trial performance compared to a no-rinse control group, which questioned the findings of studies comparing the CHO rinse with water placebo. On the other hand, Gam *et al.* [18] found similar exercise performance with CHO rinse and no-rinse conditions in contrast to the finding of Che Muhammed *et al.* [33]. Differences between the findings of these studies [18,33] might be attributed to the differences in the nutritional state of the participants (postprandial *vs*. Ramadan fasting) and the testing environmental conditions (neutral *vs*. hot-humid). As mentioned previously, there is evidence that CHO rinse is more effective when the participants fasted [10,11,18] and in more challenging environmental conditions [33]. In this regard, Lane *et al.* [11] observed increased average power outputs with a 10% CHO solution after an overnight fast whereas no improvements were observed in the fed state (*i.e.*, 2 h after a meal) [11]. On the other hand, Beelen *et al.* [17] observed that mouth rinsing with an isotonic (6.4%) MD solution in a postprandial state did not improve performance time for a 1-h cycling time trial compared with water rinse placebo. Hence, the effect of placebo rinse type (water rinse or artificially sweetened non-CHO) on exercise performance seems to be dependent on the other factors (*i.e.*, dietary state, hydration level, environmental conditions).

In the present study, mean power output values were similar between the trials indicating that it was not affected by mouth rinsing with any MD solution (3%, 6%, and 12%) compared to the placebo, which is in agreement with the findings of Beelen *et al.* [17]. Analysis of the mean power output for 5 km distance periods revealed also no differences between trials. In contrast with this result, Carter *et al.* [7] reported that power output was significantly higher in the first three quarters with CHO compared with placebo. However, in accordance with our findings, some CHO rinse studies observed no differences in power output in periods between the trials [12,17].

Physiological (heart rate, blood lactate and glucose levels) and subjective (RPE) responses to the trials were similar between maltodextrin and placebo mouth rinse. Heart rate, blood lactate, glucose and RPE increased throughout the trials (Figure 3 and Figure 4). Most studies that investigated effects of CHO mouth rinse on performance indicated that heart rate increased in response to exercise independently from CHO mouth rinse [7,16,17]. This was observed in the present study with no differences between the trials. Pottier *et al.* [12] showed a significantly higher blood lactate with isotonic CHO rinse trials compared with placebo, while blood glucose did not differ between the trials. Our findings of no differences in blood lactate and blood glucose are consistent with other studies [11,12,20] that have investigated effects of CHO mouth rinse on endurance performance.

In the present study, RPE values increased during the 20 km time trials with no maltodextrin concentration-dependent effect. Chambers *et al.* [8] provided the evidence that mouth rinse with CHO solutions containing glucose or maltodextrin activated reward related areas in brain which causes improvements in exercise performance. In that study [8], no differences were observed for RPE values between conditions although in the CHO condition there was increased power output and decreased performance time. Similar with our findings, Beelen *et al.* [17] also reported that RPE values increased in response to exercise but did not differ between the trials. Consistency in physiological [7,11,12,16,17,20] and subjective responses [8,20] to the trials in the present study and in agreement with relevant literature was considered in support of our methodology.

Similarly pacing strategy as evidenced by the trend in the changes of power output and time to complete every 5 km distance during the trial was in agreement with the literature [18,20] showing that the power output increases and the completion time decreases toward the end of the trial.

One of the factors thought to be responsible for the efficacy of CHO mouth rinse on exercise performance is the prefeeding status of the participants [29,32]. Some mouth rinse studies observed that an ergogenic effect of CHO occurred after overnight fasting [8,13] or in a post-absorptive (>4 h) state [7]. Beelen *et al.* [17] reported that 2–3 h after consumption of CHO rich meals, CHO mouth rinse did not improve exercise performance. Furthermore, Whitham and McKinney [19] indicated that CHO mouth rinse did not increase running distance after a 4 h fasting period while Pottier *et al.* [12] observed an ergogenic effect of CHO mouth rinse on exercise performance after 2 h fasting period. Nevertheless, the fasting status before exercise could influence the central neural responses to the presence of CHO in the mouth. Haase *et al.* [9] investigated cortical responses to the presence of sucrose in the mouth after 12 h fasting period or consumption of 700 kcal liquid meal with fMRI. After the 12 h fasting period, more brain regions were activated by sucrose compared with the fed state. Furthermore, these brain areas were unresponsive to artificial sweetener (saccharin) [9]. Central responses to CHO in the mouth could vary according to the prandial status prior to exercise. However, studies that investigated the effects of CHO mouth rinse on exercise performance after an overnight fast or 2 h fasting period reported that exercise performance improved with CHO mouth rinse for both fasting periods [10,11]. In our study, fasting duration was 10 h. Therefore, fasting duration likely did not affect the absence of an ergogenic effect of CHO mouth rinse in our study.

Another factor that could be accounted for the nonsignificant findings among the trials is sample size. However, the sample size ranges from 7 to 16 in corresponding studies in the literature. Power analysis revealed that in order for a medium effect size (Cohen’s *d* = 0.5) to be detected (90% chance) as significant at the 5% level, a sample of 9 participants was required in the present study. Therefore, it is unlikely that failure to find no difference among the trials was attributable to a small sample size. In addition, the absence of a trend in the data for an effect of the maltodextrin solutions may indicate that the coefficient of variation for a 20 km cycle time trial [34] was not masking potential effects.

Considering the methodology of our study, we do not have an explanation for not finding performance improvement in 20 km cycling time trial with MD rinse after an overnight fast. Further studies on the mechanisms and the confounding factors on the effectiveness of CHO rinse for endurance performance are required.

## 5. Conclusions

In conclusion, we showed that mouth rinsing with different concentrations of maltodextrin solutions did not improve performance time of 20 km cycling exercise in recreationally active males. Average power, heart rate, RPE, blood lactate and blood glucose concentrations were not affected by mouth rinsing with different concentrations of MD compared with placebo.

## Figures and Tables

**Table 1:** Nutritional intake of the participants 24 h before the trials (values were presented per kg of body weight).

	Placebo		3% MD		6% MD		12% MD		*F*	*p*	Partial η^2^
	Mean	SD	Mean	SD	Mean	SD	Mean	SD			
TEI (kcal·kg^−1^)	29.68	6.11	28.51	5.46	27.20	6.66	29.91	7.87	0.377	0.770	0.045
CHO (g·kg^−1^)	2.99	0.96	3.06	0.78	3.14	0.92	2.95	0.60	0.116	0.950	0.014
Fat (g·kg^−1^)	1.40	0.46	1.26	0.38	1.10	0.33	1.40	0.55	0.948	0.433	0.106
Protein (g·kg^−1^)	1.20	0.46	1.17	0.31	1.12	0.25	1.35	0.72	0.553	0.608	0.065
Water (mL·kg^−1^)	29.61	15.22	25.02	14.99	31.30	19.58	29.31	11.69	0.465	0.710	0.055

MD: maltodextrin; CHO: carbohydrate; TEI: total energy intake.

## Abbreviations

The following abbreviations are used in this manuscript:

CHO	carbohydrate
HR_max_	maximum heart rate
MD	maltodextrin
RPE	rating of perceived exertion
rpm	revolutions per minute
$\dot{V}O_{2max}$	maximum oxygen consumption
W_max_	maximum cycling power
